# Follow-Up of Infants Diagnosed with HIV — Early Infant Diagnosis Program, Francistown, Botswana, 2005–2012

**Published:** 2014-02-21

**Authors:** Catherine Motswere-Chirwa, Andrew Voetsch, Lydia Lu, Victor Letsholathebe, Phenyo Lekone, Esther Machakaire, Keitumetse Legwaila, Stembile Matambo, Maruping Maruping, Thatayotlhe Kolobe, Chipo Petlo, Refeletswe Lebelonyane, Mary Glenshaw, Helen Dale, Margarett Davis, Shenaaz El Halabi, Andrew Pelletier

**Affiliations:** 1 Division of Global HIV/AIDS, Center for Global Health, CDC; 2 Botswana Ministry of Health

The 2011 prevalence of human immunodeficiency virus (HIV) among pregnant women in Botswana was 30.4%. High coverage rates of HIV testing and antiretroviral prophylaxis have reduced the rate of mother-to-child transmission of HIV in Botswana from as high as 40% with no prophylaxis to <4% in 2011. In June 2005, the national Early Infant Diagnosis (EID) Program began testing HIV-exposed infants (i.e., those born to HIV-infected mothers) for HIV using polymerase chain reaction (PCR) at 6 weeks postpartum (1). During 2005–2012, follow-up of all HIV-infected infants diagnosed in all 13 postnatal care facilities in Francistown, Botswana, was conducted to ascertain patient outcomes. A total of 202 infants were diagnosed with HIV. As of September 2013, 82 (41%) children were alive and on antiretroviral therapy (ART), 79 (39%) had died, and 41 (20%) were either lost to follow-up, had transferred, or their mothers declined ART. Despite success in preventing mother-to-child transmission in Botswana, results of the EID program highlight the need for early diagnosis of HIV-infected infants, prompt initiation of ART, and retention in care.

The Botswana Prevention of Mother-to-Child Transmission (PMTCT) Program began nationwide in November 2001. Health care, including antenatal care, PMTCT services, and ART is free for Botswana citizens. Over 95% of pregnant women in Botswana register for antenatal care, and nearly all women are offered PMTCT services. Infants of HIV-positive women are tested at age 6 weeks or at first health-care contact thereafter to diagnose infections that occur during pregnancy or delivery using PCR testing on dried blood spot specimens. Breastfed infants are retested 6 weeks after breastfeeding cessation to diagnose HIV infections that might have been transmitted through breast milk. In 2011, 98% of women who received antenatal care were tested for HIV, 93% of HIV-positive pregnant women received antiretroviral prophylaxis to prevent mother-to-child transmission, and the rate of HIV infection among infants aged <18 months tested by PCR was <4% (Botswana Ministry of Health, unpublished data, 2013).

HIV-infected infants from all 13 postnatal care facilities that collect dried blood spots for EID testing were identified by PCR at Nyangabgwe HIV Reference Laboratory in Francistown, Botswana, during June 2005–December 2012. Reporting of HIV test results and referrals to treatment was determined from clinic infant testing registers. Dates for ART evaluation and initiation for infected infants were determined from the electronic register at the Infectious Disease Care Clinic in Francistown, the primary referral point for all pediatric HIV patients. Patient identifiers including first name, last name, date of birth, and sex were used to link infants across different databases. Clinical records were reviewed for patient outcomes through September 2013.

PMTCT identified a total of 10,923 HIV-exposed infants. Of these, 7,772 (71%) were tested for HIV, and 202 (2.6%) were diagnosed with HIV infection. Of the 202 HIV-infected infants, the mothers of 153 (75%) had post–HIV test counseling, and 123 (60%) infants had received ART (Figure). The median time from birth to EID testing was 9 weeks (interquartile range [IQR] = 6–23 weeks), from dried blood spot specimen collection to post-test counseling was 4 weeks (IQR = 3–7 weeks), and from post-test counseling to ART initiation was 3 weeks (IQR = 1–9 weeks). The median time from birth to ART initiation was 23 weeks (IQR = 15–48 weeks).

Through September 2013, a total of 82 (41%) children were alive and on ART (Figure). Of the 79 (39%) HIV-infected children who died, 56 died before receiving ART, and 23 died after being started on ART. Measured in person-years, the overall mortality rate for the 202 HIV-infected infants was 12.7 per 100 person-years (95% confidence interval [CI] = 10.0–16.1). The mortality rate among HIV-infected patients not receiving ART was 75.2 per 100 person-years (CI = 56.1–100.7), and the rate among patients receiving ART was 4.6 per 100 person-years (CI = 3.0–7.0). The mortality rate ratio comparing those who did not receive ART with those who received ART was 16.5 (CI = 9.8–27.7).

Fifty of the 79 patients who died had a documented cause of death. The leading causes of death were pneumonia (25 patients), gastroenteritis (nine), and sepsis (three). A total of 24 (12%) infants were lost to follow-up and 16 (8%) transferred out of the catchment area. One (0.5%) family declined ART for its infant.

## Editorial Note

Effective pediatric HIV treatment requires early diagnosis, prompt initiation of ART, and frequent monitoring to ensure retention and quality care. ART initiation in the first 3 months of life can reduce mortality by 76% (2). Without ART, 52% of perinatally HIV-infected infants and 26% of postnatally HIV-infected infants will die within 12 months (3).

Despite being among the most successful PMTCT programs in sub-Saharan Africa, the scale-up of EID in Botswana reveals several challenges in treating pediatric HIV. Almost one third of HIV-exposed infants in Francistown were not tested by the EID program. One important barrier to EID testing is that new mothers in Botswana traditionally move to their home villages after delivery, making it difficult to locate HIV-exposed infants. Other issues that might inhibit enrollment in care include fear of disclosure of their HIV status and possible stigmatization, lack of parental understanding of the need to enroll the infant in care, long wait times at clinics, and lack of transportation (4).

The results in Botswana were similar to those observed in other sub-Saharan countries. EID program data in several African countries show the estimated completion rate from HIV testing to initiation of ART ranged from 0.5% to 52.8% (5). In Tanzania, 88% of 4,292 HIV-exposed infants were tested through the EID programs in three districts; 69% of HIV infants diagnosed with HIV were enrolled in care, and 39% of infants who had received ART were retained in care (6). In a large referral hospital in South Africa, 72% of 838 HIV-exposed infants were tested through the EID program, and 67% of mothers received the HIV test results (7). Of the 38 infants diagnosed with HIV in South Africa, 61% received ART, and 34% were retained in care at 68 weeks (7).

In Botswana, lack of coordination across HIV services resulted in delays between HIV testing, post-test counseling, and ART initiation. Overall, the median time from birth to ART initiation was >5 months, including 1 month between HIV diagnosis and post-test counseling of mothers. The use of mobile phone technology has reduced the turn-around time for return of EID results to health-care providers in Zambia (8). In Botswana, electronic registries for PMTCT services and ART are being introduced in clinics to facilitate better coordination between the programs and improve outcomes for the mother and infant.

Death rates in Botswana were significantly higher among infants who did not receive ART compared with those who did. However, 19% of the 123 infants who did receive ART died by the end of the follow-up period. Pneumonia and diarrhea were the most common causes of death among HIV-infected children in the study in Francistown and also are leading causes of death among all children in Botswana aged <5 years. Botswana recently introduced vaccines against pneumococcal pneumonia and rotavirus to help prevent pneumonia and diarrhea in children. Breastfeeding has been shown to reduce overall child mortality and is recommended by the World Health Organization for HIV-infected mothers who are receiving ART (9). However, most HIV-exposed infants are fed formula that is provided for free by the government of Botswana.

The findings in this report are subject to at least four limitations. First, poor documentation at the 13 postnatal care facilities caused some discrepancies in data collected. For example, there were instances where infants were started on ART, but there was no documentation that their mothers were ever counseled after their infants were tested for HIV. Second, data were collected in Francistown and might not be representative of the rest of Botswana. Third, outcome data were not available for 20% of HIV-infected infants who were lost to follow-up or transferred. Finally, existing information management systems were not able to provide complete data on services received by infants who were examined outside of Francistown. This might have resulted in an underestimate of the percentage of HIV-exposed infants tested in the EID program.

Strategies for increasing EID testing coverage, prompt referral of HIV-diagnosed infants for ART, and retention in care are needed to ensure the survival of children who are born with HIV. These strategies include strengthening the referral system to reduce the time at each step of the route from prenatal and postnatal care to EID testing and pediatric treatment. In addition, program challenges such as educational, cultural, and structural barriers among mothers also must be addressed as part of a comprehensive EID strategy.

What is already known on this topic?

Despite high rates of human immunodeficiency virus (HIV) testing and antiretroviral prophylaxis for HIV-infected pregnant women in Botswana, up to 4% of their infants are born HIV-infected. Without antiretroviral therapy (ART), half of HIV-infected infants will die in the first year of life. Delays in HIV screening, return of test results, referral, and initiation of ART, and loss to follow-up increase infant mortality.

What is added by this report?

The Botswana Early Infant Diagnosis (EID) Program was introduced in 2005 to screen infants for HIV beginning at age 6 weeks. In Francistown, Botswana, during 2005–2012, 71% of infants born to HIV-infected women were screened for HIV. As of September 2013, among the 202 HIV-infected infants identified by the EID program, 41% were alive and on ART, 39% had died, and 20% were lost to follow-up, had transferred, or the family had declined therapy.

What are the implications for public health practice?

Even in a successful program to prevent mother-to-child transmission, survival of HIV-infected infants is poor without early diagnosis, rapid initiation of treatment, and retention in care. Strategies to overcome educational, cultural, and structural barriers are needed to improve patient outcomes in the EID program.

## Figures and Tables

**FIGURE:**
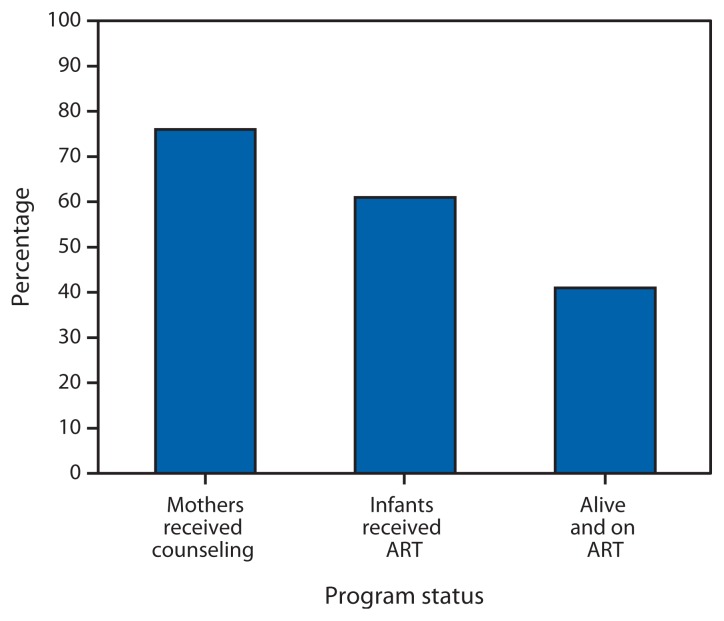
Percentage of infants diagnosed with HIV (N = 202*) whose mothers received post–HIV test counseling, percentage who received ART, and percentage who were alive and on ART through September 2013 — Early Infant Diagnosis Program, Francistown, Botswana, 2005–2012 **Abbreviations:** HIV = human immunodeficiency virus; ART = antiretroviral therapy. * A total of 10,923 infants in the program were exposed to HIV (i.e., born to HIV-infected mothers); 7,772 were tested for HIV infection, and 202 were diagnosed with HIV infection.
